# (–)-Epicatechin Supplementation Inhibits Aerobic Adaptations to Cycling Exercise in Humans

**DOI:** 10.3389/fnut.2018.00132

**Published:** 2018-12-21

**Authors:** Neil A. Schwarz, Zachary J. Blahnik, Srihari Prahadeeswaran, Sarah K. McKinley-Barnard, Shelley L. Holden, Andy Waldhelm

**Affiliations:** ^1^Department of Health, Kinesiology, and Sport, University of South Alabama, Mobile, AL, United States; ^2^Department of Physical Therapy, University of South Alabama, Mobile, AL, United States

**Keywords:** myostatin, succinate dehydrogenase, oxygen consumption (VO_2_), polyphenols, flavanols, cocoa extract, Wingate anaerobic power test, antioxidant

## Abstract

The purpose of the study was to determine if cycling exercise combined with (–)-epicatechin supplementation was more effective at increasing training adaptations than cycling combined with a placebo. Blood and muscle samples were obtained at rest before and after training to determine the effects of (–)-epicatechin supplementation on total serum antioxidant capacity, skeletal muscle mitochondrial protein content, and skeletal muscle myostatin gene expression. Participants (*n* = 20) completed two testing sessions separated by 4 weeks of cycle training, with supplementation of 100 mg (200 mg total daily) of (–)-epicatechin or a placebo, twice daily. Data were analyzed using a two-way mixed model ANOVA for each variable and the alpha level was set at *p* ≤ 0.05. A significant increase was observed for time for relative peak anaerobic power (*p* < 0.01), relative anaerobic capacity (*p* < 0.01), and fatigue index (*p* < 0.01). A significant increase was observed for time for absolute peak VO_2_ (*p* < 0.01) and peak power output obtained during the peak VO_2_ test (*p* < 0.01). A significant interaction between group and time for relative peak VO_2_ was observed (*p* = 0.04). Relative peak VO_2_ significantly increased over time in the placebo group (*p* < 0.01), but not in the (–)-epicatechin group (*p* = 0.21). A significant increase was observed for time for total serum antioxidant capacity (*p* = 0.01). No interaction or main effect of time was observed for myostatin (*p* > 0.05). Likewise, no interaction or main effect of time was observed for cytochrome C or citrate synthase (*p* > 0.05). A significant interaction effect was observed for succinate dehydrogenase (SDH; *p* = 0.02). SDH content increased significantly for the placebo group (*p* = 0.03, partial η^2^ = 0.59), but not for the (–)-epicatechin group (*p* = 0.81). Further, whereas no difference existed between the groups for SDH at baseline (*p* = 0.23), SDH content was significantly greater in the placebo group at the post time point (*p* = 0.01). Results indicate that (–)-epicatechin supplementation does not affect myostatin gene expression or anaerobic training adaptations but inhibits aerobic and mitochondrial SDH adaptations to cycle exercise training.

## Introduction

Catechin is a common flavanol found in a variety of foods and contains two stereogenic carbons and, thus, can exist as four distinct stereoisomers, despite each having the same chemical formula: (+)-catechin, (–)-catechin, (+)-epicatechin, and (–)-epicatechin (1). Of these, (–)-epicatechin is the most abundant flavanol found in and absorbed from dark chocolate, and is thought to exhibit health-promoting biological activity (1). Additionally, (–)-epicatechin was demonstrated to be the only catechin stereoisomer capable of inducing vasodilation of the femoral artery upon direct infusion into the bloodstream. However, (–)-epicatechin undergoes substantial metabolism into structurally related (–)-epicatechin metabolites before entering the circulation, which may or may not alter its function (1–5).

Results from human trials indicate that (–)-epicatechin elicits beneficial effects on the vascular system (6, 7). Acute administration of 200 mg of (–)-epicatechin resulted in the augmentation of nitric oxide production and reduced endothelin-1, a marker of oxidative stress, in healthy men (6). Similar results were reported for nitric oxide production in healthy males after ingestion of (–)-epicatechin-rich cocoa (7). In addition to its potential beneficial effects on endothelium and vascular function, (–)-epicatechin demonstrates antioxidant properties. In one study, an increase in plasma epicatechin was associated with an increase in plasma antioxidant capacity and a concomitant decrease in plasma oxidation products (8). As a flavonoid with a complex structure, as well as being metabolized before entering the circulation, it is difficult to describe the exact mechanism of action through which (–)-epicatechin acts as an antioxidant. It is possible that (–)-epicatechin acts as an antioxidant both directly as a scavenger of free radicals and indirectly as a modulator of superoxide dismutase and glutathione peroxidase (9). (–)-epicatechin has also been demonstrated to modulate macronutrient metabolism in normal and overweight subjects (10). Oral (–)-epicatechin supplementation at a dose of 1 mg/kg of bodyweight decreased the respiratory quotient after meal consumption, suggesting a higher rate of fat oxidation. In patients with type 2 diabetes and heart failure, 100 mg per day of (–)-epicatechin for 3 months increased peroxisome proliferator-activated receptor γ coactivator-1α (PGC-1α), silent mating type information regulation 2 homolog (SIRT1), and mitochondrial transcription factor A (Tfam) (11). Consequently, mitochondrial structure was enhanced, although an increase in mitochondrial quantity was not observed. In another study, (–)-epicatechin administration reduced myostatin and increased markers of skeletal muscle myogenesis in both young and old mice (12). Furthermore, in humans, ingestion of 1 mg/kg of bodyweight of (–)-epicatechin for 7 days increased bilateral grip strength by roughly 7% and demonstrated a favorable change in the follistatin-to-myostatin ratio (12).

Because of the aforementioned observations with (–)-epicatechin supplementation, it has been suggested that it may be useful as a means to increase exercise adaptations (13). Fifteen days of (–)-epicatechin supplementation alone resulted in increased exercise performance, reduced muscle fatigue, increased muscle capillarity and increased mitochondrial biogenesis in mice (14). When combined with exercise, additional increases were observed (14). In mice selectively bred for low running capacity, 30 days of (–)-epicatechin supplementation successfully increased skeletal muscle capillarity and mitochondrial biogenesis (15). In another study, (–)-epicatechin supplementation in mice, along with treadmill exercise over the course of 8 weeks, increased angiogenesis and mitochondrial biogenesis (16). Additionally, the distance achieved on the treadmill was increased by 84% in the (–)-epicatechin supplementation and exercise group, whereas exercise alone and (–)-epicatechin supplementation alone resulted in an increase of 69 and 46%, respectively. All groups, except for the control, increased performance with the greatest magnitude of increase observed in the (–)-epicatechin supplementation and treadmill exercise group (16). In contrast, 24 days of (–)-epicatechin supplementation in rodents did not increase exercise endurance capacity, VO_2_ peak, muscle blood flow, or vascular conductance; however, mean arterial pressure was decreased, suggesting a mild improvement in cardiovascular function but with no transfer to exercise performance (17).

In humans, acute dark chocolate consumption prior to prolonged exercise resulted in lower oxidative stress, but did not affect immunoendocrine status (18). Researchers reported ~96 mg of epicatechin in the chocolate, but the exact stereoisomer was not reported (+ or –). Other researchers found no benefit to ingesting flavanol-containing (specific flavanols not reported) cocoa beverages on indices of muscle damage after downhill running (19). Despite promising evidence in rodent models, the paucity of evidence involving (–)-epicatechin supplementation in humans subjects in combination with exercise training is insufficient for interpretation as to if and how flavanols modulate exercise adaptations. Thus, the purpose of this study was to investigate the effects of 4 weeks of (–)-epicatechin supplementation in conjunction with aerobic and anaerobic exercise training on aerobic and anaerobic power output, serum antioxidant capacity, skeletal muscle mitochondrial protein content, and skeletal muscle myostatin mRNA expression in humans. It was hypothesized that (–)-epicatechin supplementation combined with cycling would improve measures of aerobic and anaerobic fitness, increase total serum antioxidant capacity, increase skeletal muscle mitochondrial protein content, and decrease skeletal muscle myostatin mRNA expression more than cycling combined with placebo.

## Materials and Methods

### Participants

A total of 29 recreationally-active men and women (exercised for 30 min at least twice per week, on average, for at least the past 6 months) between 18 and 30 years of age [(–)-epicatechin (EPI) group: 20.5 ± 1.5 years.; placebo (PLA) group: 21.0 ± 1.9 years.] were recruited to participate in this study. Participants considered as a low risk for cardiovascular disease, as outlined by the American College of Sports Medicine (ACSM), and who were not consuming nutritional supplements deemed an ergogenic aid for exercise performance, were allowed to enter the study. Exclusion criteria included prior lower extremity surgeries or indication of current or past health conditions, as noted on a health history questionnaire, which limited participation in exercise. The study was approved by the University of South Alabama Institutional Review Board. All participants provided written informed consent prior to enrollment after receiving both oral and written information about the study. All experimental procedures involved in the study conformed to the ethical consideration of the Declaration of Helsinki.

**Figure 1:**
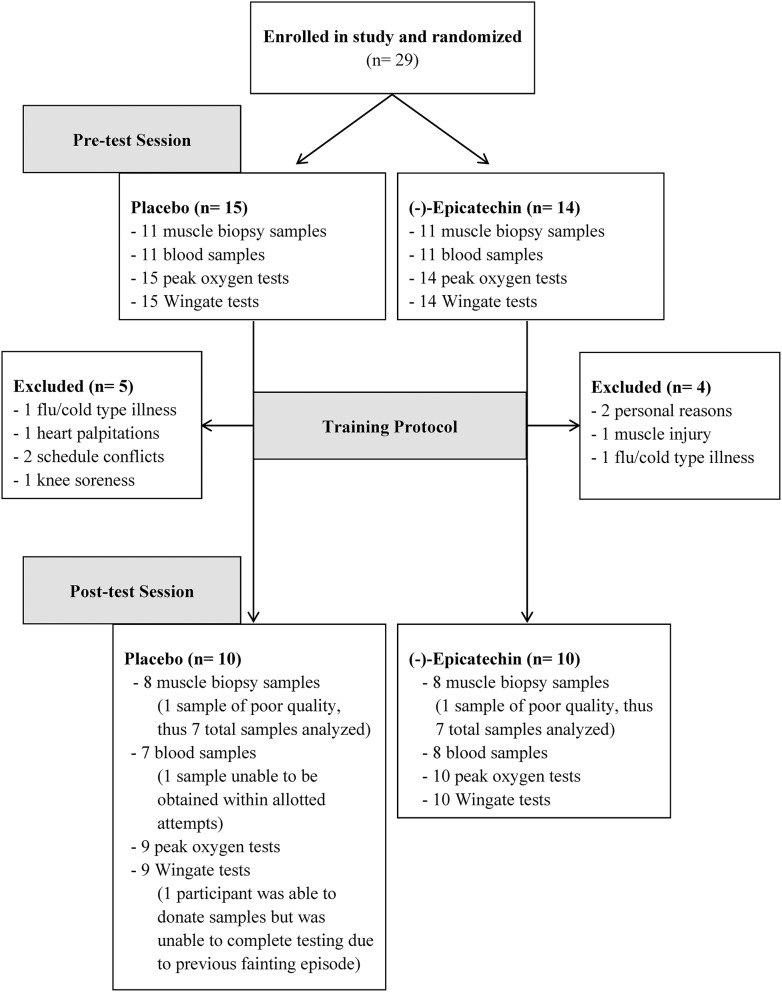
Participant flow chart.

Overall, 20 of the 29 participants completed the study. Of the 9 participants that withdrew from the study; reasons for withdrawal included muscle injury (1), knee soreness (1); flu/cold type illnesses (2), heart palpitations [1; placebo group], family/schedule conflicts (2), and personal reasons (2). One participant experienced a hypoglycemic fainting episode after the last training session (placebo group), and thus did not participate in the post-training exercise testing for safety reasons; however, the participant still donated post-testing blood and muscle samples. Another participant was unable to complete the post-exercise peak oxygen consumption test because of equipment malfunction. Additionally, only 22 of the original 29 participants agreed to donate blood and muscle samples, and 16 of these participants completed the entire study. See Figure 1 for a flow diagram of participants and Table 1 for participant characteristics for those who completed the entire study at baseline.

**Table 1:** Participant Baseline Characteristics.

**Variable**	**Mean ± *SD***
**ANTHROPOMETRICS**	
Height (cm)	172.8 ± 10.3
Body Mass (kg)	76.1 ± 18.8
**HEMODYNAMICS**	
Resting Heart Rate (bpm)	70.5 ± 14.8
Systolic Blood Pressure (mmHg)	123.6 ± 9.6
Diastolic Blood Pressure (mmHg)	74.2 ± 7.7
**PEAK OXYGEN CONSUMPTION TEST**	
Relative VO_2_ Peak (ml/kg/min)	33.8 ± 9.5
Absolute VO_2_ Peak (L/min)	2.6 ± 0.9
Peak Power Output (W)	212.2 ± 60.4
**WINGATE ANAEROBIC CYCLE TEST**	
Relative Peak Power Output (W/kg)	15.3 ± 1.9
Relative Anaerobic Capacity (W/kg)	7.8 ± 1.8
Fatigue Index (W/s)	28.8 ± 8.9

### Experimental Design

This study utilized a double-blind, randomized, placebo-controlled parallel design. Participants were randomly assigned to the EPI group or PLA group while completing a 4 weeks anaerobic and aerobic cycle training protocol. Participants completed a familiarization session, a pre-testing session, a cycle training protocol, and a post-testing session during the course of the study.

### Supplementation Protocol

The EPI group consumed one capsule containing 100 mg of 98% pure (–)-epicatechin twice daily (200 mg total). Participants were instructed to consume one 100 mg capsule in the morning and one 100 mg capsule in the afternoon or evening. The PLA group consumed identical looking capsules containing 100 mg of cellulose twice daily. The supplement and placebo were manufactured and blinded by Vital Pharmaceuticals Inc. (VPX Inc., Weston, FL).

### Familiarization Session

The familiarization session was primarily used to introduce the participants to the exercise protocol and to the exercise and testing instrumentation. Participants‘ height was measured using a stadiometer (Seca, Chino, CA). The participants sat on each cycle ergometer to be used for testing and the seat was adjusted to the appropriate height so that knee flexion was ~20–25 degrees at full extension. The participant cycled against no resistance for a short period to confirm the correct seat height. The seat position was recorded for use during the testing and exercise sessions. During this session, each participant performed a practice trial for the Wingate anaerobic cycle test to become familiar with the demands of the test.

### Pre- and Post-testing Sessions

The pre- and post-testing sessions were conducted in identical fashion. Participants did not eat or drink liquids, except water, for 10 h prior to each testing session. Participants refrained from strenuous exercise for at least 48–72 h before each testing session. Participants reported to the Human Performance Lab at a pre-scheduled time in the morning. Upon arrival participants were weighed and then, after 5 min of seated rest, heart rate and blood pressure were assessed in the seated position. Next, venous blood and muscle biopsy samples were obtained, after which the participants performed a 30 s Wingate anaerobic cycle test. Following the Wingate test, the participants rested for 15 min. After resting, the participants performed a peak oxygen uptake assessment using a cycle ergometer. Post-testing sessions were completed between 48 and 72 h after the final training session. Supplementation was discontinued 24 h before the post-testing session.

### Wingate Anaerobic Cycle Test

Participants warmed-up for 5 min at a work rate equal to 75 Watts on the Velotron cycle ergometer (Racer-Mate, Seattle, WA). After the 5 min warm-up period, subjects continued to warm-up for an additional 20 s followed by a 6 s acceleration phase, during which they pedaled as fast as possible against no resistance to attain peak cadence. Immediately at the end of this phase, a load equal to 7.5% of bodyweight was applied to the flywheel and subjects pedaled as fast as possible for 30 s. Data was recorded and saved using the Velotron Wingate software (Racer-Mate, Seattle, WA).

### Peak Oxygen Uptake Assessment

Participants performed a maximal oxygen consumption exercise test on an electromagnetically controlled cycle ergometer (Ergometrics 800, Sensormedics, Yorba Linda, CA) to determine their peak oxygen consumption (VO_2_ peak). Oxygen uptake during the test was measured via an open-circuit sampling system (Vmax Encore 29C, Carefusion, Yorba Linda, CA), and the highest level of VO_2_ obtained for 1 min was defined as VO_2_ peak. Respiratory gas exchange was measured by having the participant wear a facemask that was connected to the Encore 29C system. The load on the bike was increased incrementally so that power output increased 25W per minute. The exercise test was performed until the participant was no longer able to maintain a cycling cadence above 40 revolutions per minute.

### Training Sessions

Participants engaged in four cycling exercise sessions per week for 4 weeks for a total of 16 sessions (Table 2). Sessions one and three of each week were completed independently at the University of South Alabama Recreation Center using the prescribed intensity and duration and completion of each session was reported to study personnel. For the training sessions in the recreation center, participants used the True Fitness CS 800 upright bicycle (True Fitness, St. Louis, MO). The work rate and duration used at the Recreation Center and how to program the bike was explained in detail and given in writing. If needed, a study personnel member met the participant at the recreation center for the first session to help with correct programming of the bike for the training sessions. Sessions two and four of each week were performed in the Human Performance Laboratory in the Health, Kinesiology, and Sport Building using the Ergometrics 800 (Sensormedics, Yorba Linda, CA) and Velotron (Racer-Mate, Seattle, WA) cycle ergometers, respectively. Participants who completed the study completed 100% of the training sessions. Some deviation in the rest period existed for exercise session four of each week because, in some cases, the participants needed more time to recover before the next set.

**Table 2:** Outline of Training Sessions.

	**Sessions 1 and 3**		**Session 2**				**Session 4**			
**Week**	Load^*^(%)	**Time**	Load^*^(%)	**Time**	**Sets**	**Rest**	**Load^†^(%)**	**Time**	**Sets**	**Rest**
1	50	45 min	90	60 s	6	75 s	4	20 s	4	240 s
2	50	50 min	90	60 s	8	75 s	4	25 s	5	240 s
3	50	55 min	95	75 s	8	75 s	4.5	30 s	6	240 s
4	50	60 min	95	75 s	10	75 s	5	30 s	6	240 s

* *percentage of power output (Watts) at peak oxygen uptake during pre-testing session*.

† *percentage of body mass*.

### Collection of Venous Blood and Muscle Biopsy Samples

Blood and muscle samples were collected at rest. Venous blood from the antecubital vein was collected into a serum separator tube using a Vacutainer apparatus and needle (Becton, Dickinson and Company, Franklin lakes, NJ). Immediately after blood draw, blood samples remained at room temperature for 20 min to clot, and then were centrifuged (1,000 g) for 20 min to separate serum. Serum samples were aliquoted into 1.5 mL tubes and immediately frozen at −80°C for the later analysis. Percutaneous muscle biopsies (20–25 mg) were obtained from the middle portion of the vastus lateralis muscle at the midpoint between the patella and the greater trochanter of the femur at a depth between 1 and 2 cm based on previously-used procedures (20). The same leg and general location (determined by pre-biopsy markings) was biopsied at each testing session. The biopsy area was shaved clean of leg hair and cleaned with rubbing alcohol. A small area of the cleaned skin ~2 cm in diameter was anesthetized with a 1.5 mL subcutaneous injection of 1% lidocaine HCl (Hospira, Lake Forest, IL). After, the biopsy site was further cleansed by swabbing the area with povidine-iodine. Once anesthetized, a pilot hole was created using a sterile 12-gauge needle followed by insertion of a 14-gauge fine needle aspiration biopsy instrument (Pro-Mag Ultra Automatic Biopsy Instrument, Argon Medical, Gainesville, FL) was inserted into the skin at an approximate depth of 1 cm to extract the muscle sample using three passes. After removal, adipose tissue was trimmed from the muscle specimens. Specimens were immediately immersed in 500 μL of RNA*later* stabilization solution (Life Technologies, Carlsbad, CA) and stored at −80°C for later analysis.

### Total RNA and Protein Isolation

Total RNA and protein were isolated from muscle samples using the mirVana PARIS kit according to manufacturer's specifications (Life Technologies, Carlsbad, CA). Muscle samples were thawed on ice and blotted to remove excess RNA*later* solution. Samples were added to 2.0 ml tubes pre-filled with beads and homogenized in 500 μl of ice-cold cell disruption buffer (BeadBug 3, Benchmark Scientific, Sayreville, NJ) with protease and phosphatase inhibitors added (Halt Protease and Phosphatase Inhibitor Cocktails, Thermo Scientific, Waltham, MA). After homogenization, 200 μL of lysate for RNA isolation was removed and mixed thoroughly with denaturing solution and incubated on ice for 5 min. The remaining lysate for protein analysis was incubated on ice for 15 min, clarified by centrifugation, and pipetted to a fresh tube and stored at −80°C until further use. After incubating for 5 min on ice, the lysate portion with denaturing solution for RNA isolation was mixed with acid-phenol:chloroform, vortexed for 1 min, and then centrifuged for 5 min. The top aqueous phase was removed and transferred to a fresh microtube and 100% ethanol was added and mixed thoroughly. The lysate/ethanol mixture was placed onto a filter cartridge and centrifuged for 30 s with the flow-through being discarded. The filter cartridge was then washed 3 times with the appropriate wash solution and then centrifuged to remove excess wash solution. Pre-heated RNase-free water was then centrifuged through the filter cartridge to collect the total RNA and then quantified.

### Total RNA Quantification and cDNA Synthesis

Total RNA concentration was determined spectrophotometrically (SpectraMax 384 Plus and SoftMax Pro Software, SpectraDrop Micro-Volume Microplate, Molecular Devices, San Jose, CA) by optical density (OD) at 260 nm using an OD260 equivalent to 40 μg/μl. Sample purity was checked using OD260/OD280 ratio with a result of 1.99 ± 0.10. Reverse-transcription to synthesize cDNA was performed with 100 ng of total RNA template using the qScript Flex cDNA Kit following manufacturer's specifications (Quantabio, Beverly, MA).

### PCR Amplification and Quantitation of Myostatin

Primers for glyceraldehyde 3-phosphate dehydrogenase (GAPDH) and myostatin were commercially synthesized (Integrated DNA Technologies, Coralville, IA). The forward primer sequence used for GAPDH was ACCACAGTCCATGCCATCAC, and the reverse primer sequence used was TCCACCACCCTGTTGCTGTA (21). The forward primer sequence used for myostatin was CTACAACGGAAACAATCATTACCA, and the reverse primer sequence used was GTTTCAGAGATCGGATTCCAGTAT (22). Reactions totaling 20 μL consisting of 4 μL of cDNA template, 10 μL of PerfeCta SYBR Green SuperMix (Quantabio, Beverly, MA), 0.6 μL of the reverse primer reaction mixture, 0.6 μL of the forward primer reaction mixture, and 4.8 μL of nuclease-free water were added to each well. Each reaction was amplified using real-time quantitative PCR (qTower 2.2, Analytik Jena US LLC, Beverly, MA). The amplification profile was run for an initial denaturation at 95°C for 3 min and then for 40 cycles of 95°C for 15 s and 58°C for 45 s. Fluorescence was measured after each cycle. Relative myostatin mRNA expression was determined using the 2^ΔΔCt^ method with GAPDH as the reference gene (23). Data were expressed with post-testing levels normalized to pre-testing levels for each group. The specificity of the PCR was demonstrated with an absolute negative control reaction containing no cDNA template, and a single gene product was confirmed using DNA melt curve analysis.

### Total Protein and Mitochondrial Protein Quantification

Total protein concentration was determined in triplicate using the Pierce BCA Protein Assay Kit (Thermo Scientific, Waltham, MA) with albumin as a standard. Skeletal muscle citrate synthase (CS), cytochrome C (CytC), and succinate dehydrogenase (SDH) content were determined by an enzyme-linked immunosorbent assay (ELISA; CytC: Thermo Fisher Scientific, Waltham, MA, CAT# KHO1051; CS: GBiosciences, St. Louis, MO, CAT# IT3053; SDH: GBiosciences, St. Louis, MO, CAT# IT4343) in duplicate using a microplate reader (SpectraMax 384 Plus and SoftMax Pro Software, Molecular Devices, San Jose, CA). Intra-assay coefficients of variability (CV) for CS, CytC, and SDH were 4.62, 5.20, and 11.98%, respectively. Mitochondrial protein content was expressed relative to the total protein concentration (ng/mg).

### Serum Total Antioxidant Capacity

The total antioxidant capacity of the serum samples was determined as Trolox equivalents (TE) in duplicate using a plate-based colorimetric measurement at 405 nm (SpectraMax 384 Plus, and SoftMax Pro Software, Molecular Devices, San Jose, CA) according to manufacturer specifications (Cayman Chemical; Ann Arbor, MI; CAT# 709001). The intra-assay CV for total antioxidant capacity was 6.84%.

### Statistical Analysis

Data are expressed as mean and standard deviation (*M* ± *SD*). Normality of data was assessed by the Shapiro-Wilk test and visual inspection of Q-Q plots. Statistical analyses were performed by utilizing a separate 2 × 2 (group × time point) two-way mixed model analyses of variance (ANOVA) for each criterion variable. If a group by time point interaction existed, a separate one-way ANOVA for each group and time point was performed to determine simple main effects. Effect size was calculated as partial eta-squared (η^2^). The critical value for significance was set at ≤ 0.05 for all tests. All statistical procedures were performed using the SPSS Statistics 22.0 software (IBM, Armonk, NY).

## Results

### Total Body Mass and Hemodynamics

There was no significant interaction between group and time for total body mass (*p* > 0.05). Additionally, no significant difference was observed for the main effects of group or time for total body mass (*p* > 0.05). No significant interaction between group and time for heart rate, systolic blood pressure, or diastolic blood pressure was observed (*p* > 0.05). Further, no significant differences were observed for the main effect of time (*p* > 0.05). The total body mass and hemodynamics data are found in Table 3.

**Table 3:** Total Body Mass and Hemodynamics for Each Group and Time Point.

**Variable**	**Time point**	**(–)-Epicatechin^**a**^**	**Placebo^**b**^**
Total Body Mass (kg)	Pre	76.2 ± 22.0	76.0 ± 15.8
	Post	76.5 ± 22.0	75.9 ± 15.7
Resting Heart Rate (bpm)	Pre	73.0 ± 14.9	67.7 ± 15.0
	Post	71.5 ± 13.8	67.2 ± 11.3
Systolic Blood Pressure (mmHg)	Pre	124.2 ± 7.5	122.9 ± 11.9
	Post	121.4 ± 6.0	120.4 ± 14.6
Diastolic Blood Pressure (mmHg)	Pre	74.8 ± 7.1	73.6 ± 8.7
	Post	74.8 ± 8.1	71.3 ± 6.2

a *n = 10*.

b *n = 9*.

*Data are presented as mean and standard deviation (M ± SD). No statistical significance observed*.

### Peak Oxygen Consumption

**Figure 2:**
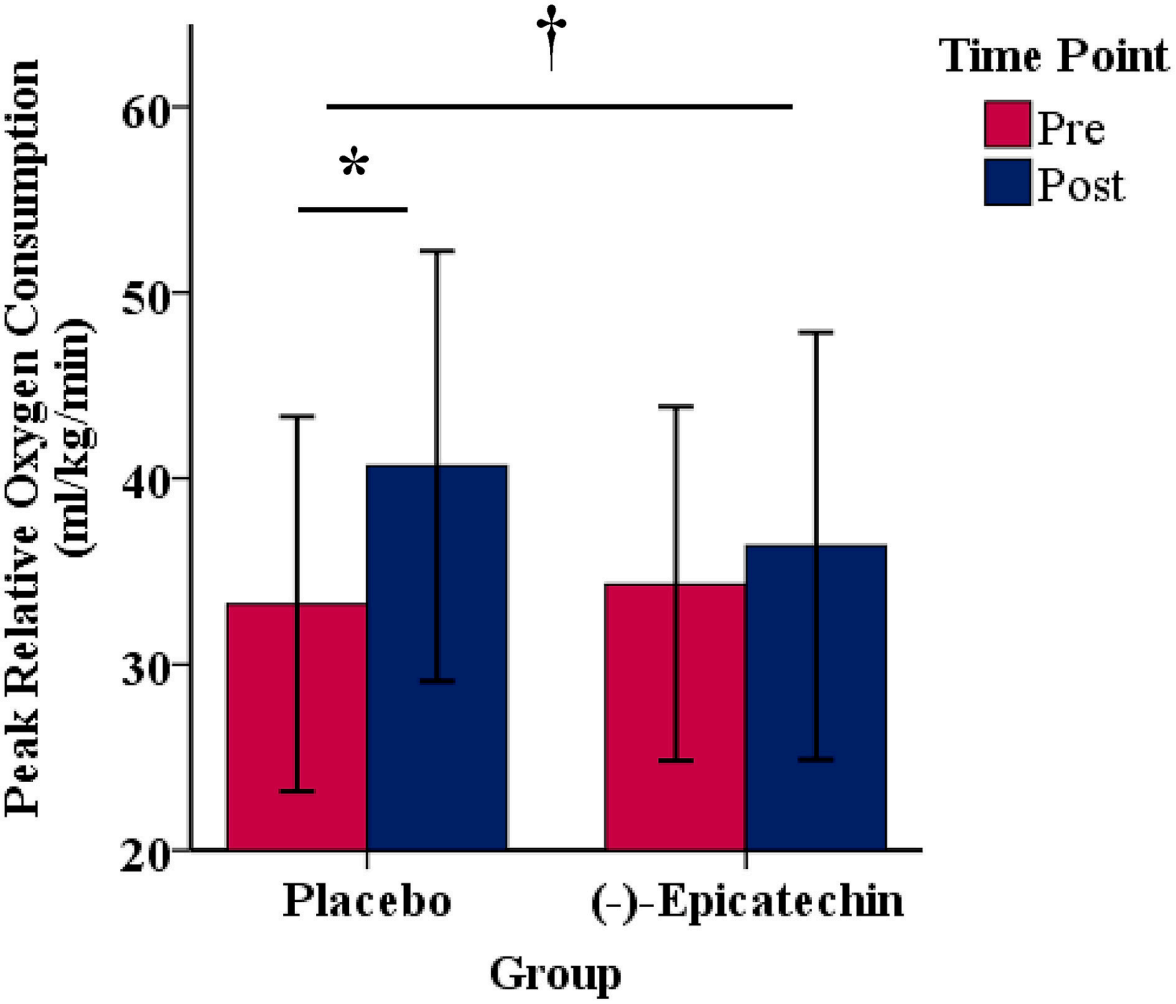
Peak relative oxygen consumption for each group and time point.denotes a significant interaction between the group and time. * denotes a significant difference between the pre and post time points. Error bars represent ±one standard deviation from the mean.

**Figure 3:**
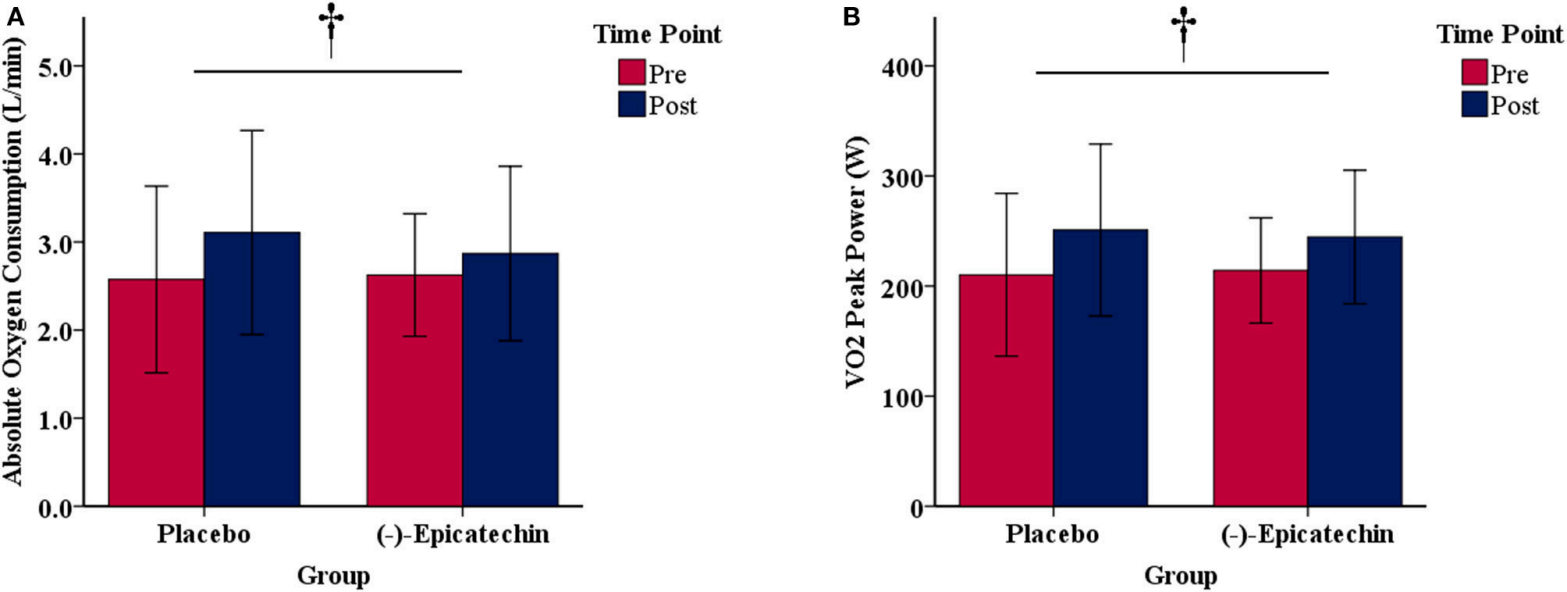
**(A)** Absolute oxygen consumption and **(B)** peak power output obtained during the peak oxygen consumption test for each group and time point.denotes a significant difference for the main effect of time. Error bars represent ±one standard deviation from the mean.

A significant interaction between group and time for relative peak VO_2_ was observed (*p* = 0.04, partial η^2^ = 0.24; Figure 2). The relative peak VO_2_ significantly increased over time for the PLA group (*p* < 0.01, partial η^2^ = 0.66), but not for the EPI group (*p* > 0.05). No statistically significant differences were observed between the groups for the pre or post time points (*p* > 0.05). There was no significant interaction between group and time for absolute peak VO_2_ (*p* > 0.05) or peak power output obtained during the peak VO_2_ test (*p* > 0.05). A statistically significant difference was observed for the main effect of time for absolute peak VO_2_ (*p* < 0.01, partial η^2^ = 0.48; Figure 3A) and peak power output obtained during the peak VO_2_ test (*p* < 0.01, partial η^2^ = 0.66; Figure 3B). All data from the peak oxygen consumption test for each group and time point are found in Table 4.

**Table 4:** Peak Oxygen Consumption Test Measurements for Each Group and Time Point.

**Variable**	**Time point**	**(–)-Epicatechin^**a**^**	**Placebo^**a**^**
Relative Peak VO_2_ (ml/kg/min)	Pre	34.3 ± 9.5	33.2 ± 10.1
	Post	36.4 ± 11.5	40.7 ± 11.6
Absolute Peak VO_2_ (L/min)	Pre	2.6 ± 0.7	2.6 ± 1.1
	Post	2.9 ± 1.0	3.1 ± 1.2
VO_2_ Peak Power Output (W)	Pre	214 ± 48	210 ± 74
	Post	238 ± 61	251 ± 78

a *n = 9*.

*Data are expressed as mean and standard deviation (M ± SD). VO_2_ = volume of oxygen consumption*.

### Wingate Anaerobic Cycle Test

**Figure 4:**
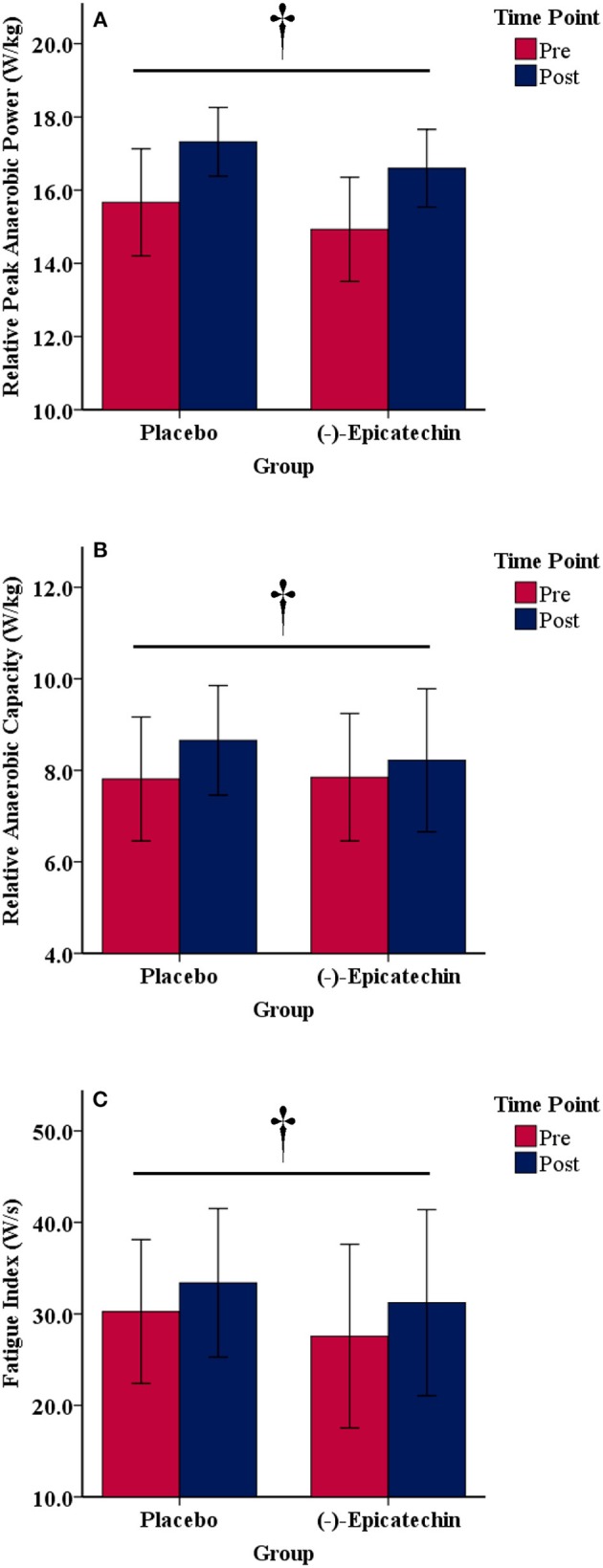
**(A)** Relative peak anaerobic power, **(B)** relative anaerobic capacity, and **(C)** fatigue index results for each group and time point.denotes a significant difference for the main effect of time. Error bars represent ±one standard deviation from the mean.

There was no significant interaction between group and time for relative peak anaerobic power, relative anaerobic capacity, or fatigue index (*p* > 0.05). A statistically significant difference was observed for the main effect of time for relative peak anaerobic power (*p* < 0.01, partial η^2^ = 0.74; Figure 4A), relative anaerobic capacity (*p* < 0.01, partial η^2^ = 0.46; Figure 4B), and fatigue index (*p* < 0.01, partial η^2^ = 0.47; Figure 4C). All data from the Wingate anaerobic cycle test for each group and time point are found in Table 5.

**Table 5:** Wingate Anaerobic Cycle Test Measurements for Each Group and Time Point.

**Variable**	**Time point**	**(–)-Epicatechin^**a**^**	**Placebo^**b**^**
Relative Peak Anaerobic Power (W/kg)	Pre	14.9 ± 2.0	15.7 ± 1.9
	Post	16.6 ± 1.5	17.3 ± 1.2
Relative Anaerobic Capacity (W/kg)	Pre	7.9 ± 2.0	7.81 ± 1.8
	Post	8.2 ± 2.2	8.66 ± 1.6
Fatigue Index (W/s)	Pre	27.6 ± 10.0	30.3 ± 7.9
	Post	31.2 ± 10.2	33.4 ± 8.1

a *n = 10*.

b *n = 9*.

*Data expressed as mean and standard deviation (M ± SD)*.

### Myostatin mRNA Expression

**Figure 5:**
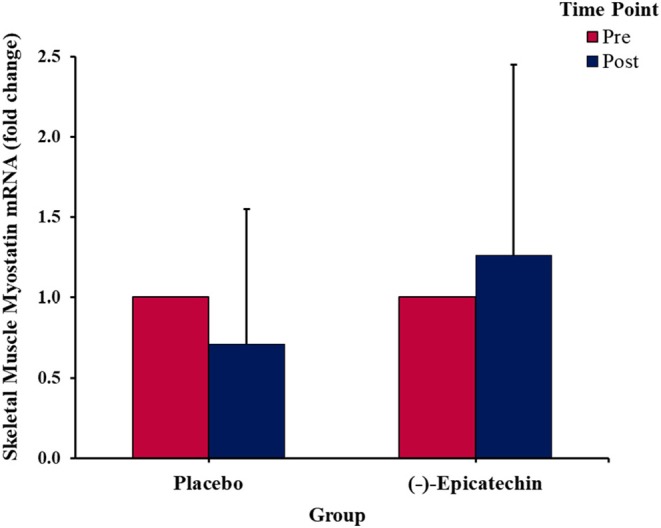
Fold change in the skeletal muscle myostatin mRNA expression. Error bars represent ±one standard deviation from the mean.

No significant interaction between group and time was observed for myostatin expression (*p* > 0.05; Figure 5). Further, no significant differences were observed for the main effect of time (*p* > 0.05).

### Mitochondrial Protein Content

**Figure 6:**
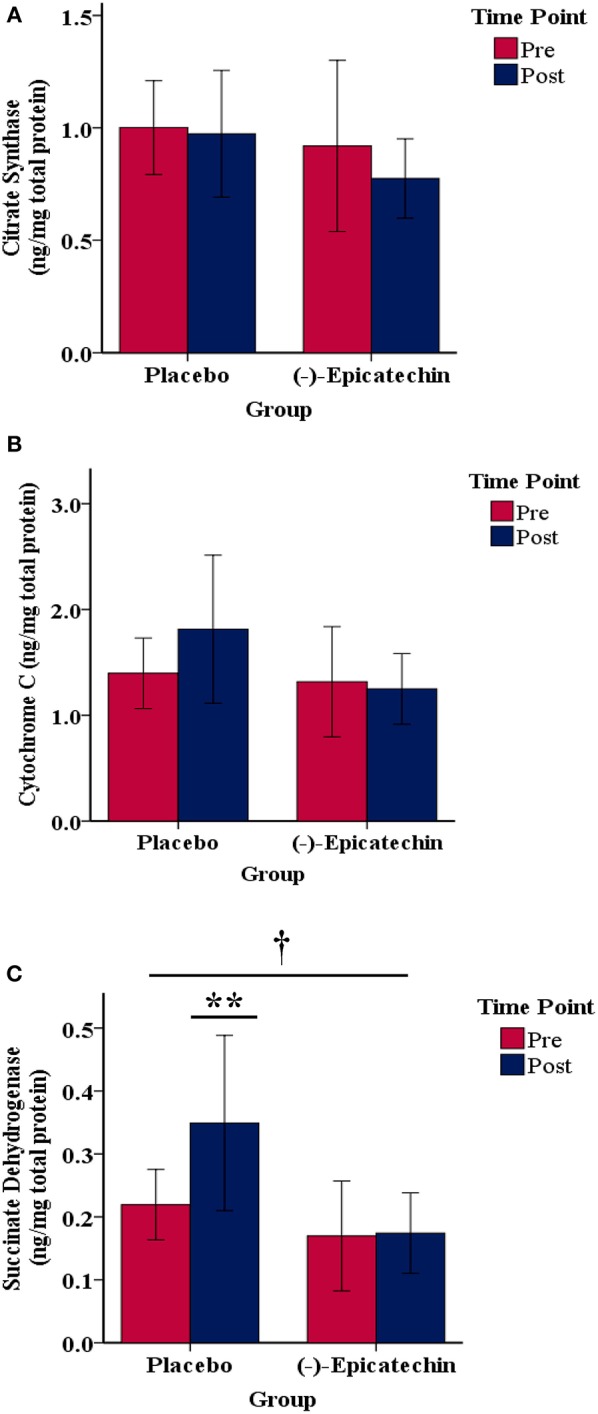
Skeletal muscle **(A)** citrate synthase, **(B)** cytochrome C, and **(C)** succinate dehydrogenase protein content relative to the total protein for each group by time point.denotes a significant interaction between the group and time. ** denotes the post time point for the placebo group is statistically greater compared with the pre time point for the placebo group and statistically greater compared with the post time point for the (–)-epicatechin group; error bars represent ±one standard deviation from the mean.

No significant interaction effect or main effect of time was observed for skeletal muscle cytC or CS content (*p* > 0.05; Figures 6A,B). A significant interaction effect was observed for skeletal muscle SDH content (*p* = 0.02; partial η^2^ = 0.36; Figure 6C). The skeletal muscle SDH content increased significantly for the PLA group (*p* = 0.03, partial η^2^ = 0.59), but not the EPI group (*p* > 0.05). Further, whereas no difference existed between the groups for SDH at baseline (*p* > 0.05), SDH content was significantly greater in the PLA group compared with the EPI group at the post time point (*p* = 0.01; partial η^2^ = 0.43).

### Serum Total Antioxidant Capacity

**Figure 7:**
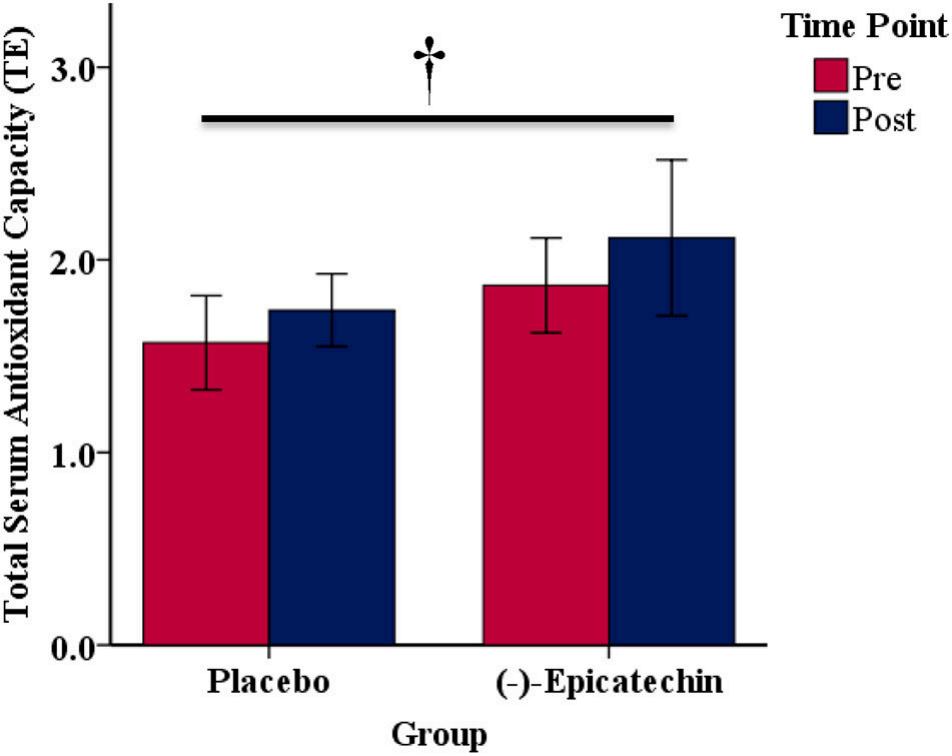
Total serum antioxidant capacity expressed as Trolox equivalents (TE) for each group and time point.denotes a significant difference for the main effect of time. Error bars represent ±one standard deviation from the mean.

No significant interaction between group and time was observed for total serum antioxidant capacity (*p* > 0.05). A significant main effect of time was observed with total serum antioxidant capacity increasing from pre to post (*p* = 0.01; partial η^2^ = 0.39; Figure 7).

## Discussion

The purpose of the current study was to determine the effect of 4 weeks of (–)-epicatechin supplementation on adaptations to anaerobic and aerobic cycling in a healthy adult population. (–)-Epicatechin has been shown, by multiple studies, to increase capillary and mitochondrial density, decrease myostatin expression and increase follistatin expression in humans (12, 24). The results of this study revealed an inhibitory effect of (–)-epicatechin supplementation on development of peak relative aerobic power and mitochondrial density as determined by skeletal muscle SDH protein content in response to cycle training. These observations were observed despite equal increases in peak power obtained during the peak oxygen consumption test. The PLA group increased relative peak VO_2_ by an average of 7.4 ± 5.7 ml/kg/min whereas the EPI group only increased by an average of 2.0 ± 4.4 ml/kg/min. This finding was in contrast to the hypothesis that (–)-epicatechin would augment aerobic adaptations. In agreement, the EPI group failed to show an increase in SDH protein content, thus potentially suggesting that (–)-epicatechin blunts increases in relative peak VO_2_ in response to training via the inhibition of mitochondrial adaptations. These findings are in contrast to the findings of studies involving rodent models (14, 15). Additionally, (–)-epicatechin conferred no additional benefit for peak anaerobic power or anaerobic capacity when compared to the supplementation with a placebo. In further contrast to earlier studies (12, 24), our results indicated no effect on myostatin gene expression after 4 weeks of supplementation.

(–)-epicatechin demonstrates antioxidant activity and; therefore, total antioxidant capacity of serum was analyzed (25, 26). The current study revealed an increase in total antioxidant capacity as a result of exercise training, but not (–)-epicatechin supplementation. In some instances, antioxidants display an ability to reduce fatigue and enhance exercise performance with acute supplementation (27). The benefit of long-term supplementation of antioxidants; however, is more controversial as some evidence suggests they may inhibit exercise adaptions as reactive oxygen species are thought to act as a molecular signal for the cellular pathways associated with mitochondrial biogenesis. However, it should be emphasized that measurement of total serum antioxidant capacity does not completely represent oxidation status, thus the results of this study should be interpreted with caution (28). The results of this study do not indicate that adaptations were potentially blunted as a result of excessive antioxidant activity; however, only the resting serum total antioxidant activity was measured. It is plausible, but not demonstrated in the current study, that (–)-epicatechin supplementation may alter acute antioxidant activity in response to each exercise bout, possibly affecting the adaptation potential over time. Paulsen et al. (29) demonstrated changes in cellular signaling in response to exercise with supplementation of vitamin C and vitamin E, both antioxidants. With results similar to this study, Ostojic et al. (30) found that coffeeberry (a strong antioxidant) supplementation negatively impacted VO_2_ max during a 4 week training study compared with a placebo. Vitamin C supplementation has previously been shown to reduce increases in cytochrome C content in response to endurance training in rodents along with blunting the signaling pathways associated with mitochondrial biogenesis and antioxidant enzyme gene expression (31). Other researchers have also reported a blunting of antioxidant enzyme production and PGC-1α as a result of antioxidant supplementation with exercise (32). PGC-1α and associated biomarkers were upregulated after (–)-epicatechin supplementation in one study (11); however, the study population included persons with type 2 diabetes and heart failure such that the results are likely not applicable to healthy populations. As for (–)-epicatechin supplementation in healthy individuals performing exercise, further research is needed to explore the mechanisms responsible for the potential blunting of aerobic training adaptations. The current study lacks mechanistic explanation for why these results were observed.

Anaerobic power and anaerobic capacity increased from training with no difference between the groups. Additionally, although there was an increase in the fatigue index over time, no group differences were observed. The change in peak anaerobic power observed in this study is similar in magnitude to a previous study (33). Vera-Ibañez et al. (33) studied the effect of high-intensity cycling for 4 weeks on peak power output during the Wingate test. They observed an increase in peak power output of ~15% whereas the current study observed an increase of ~11%. The Wingate cycle test requires a degree of learning. In the current study, it was observed that the participants were able to increase their maximum revolutions per minute (RPM) with the training sessions. Thus, when the resistance was applied at the start of the test, the initial RPM was higher and resulted in higher peak power output. Although relative anaerobic capacity increased, fatigue index also increased. These results indicate that peak power output increased to a greater degree than anaerobic capacity, and, because of this, power output dropped at a faster pace throughout the 30 s test compared to the pre-test resulting in a higher fatigue index. Even with a greater fatigue index, participants demonstrated a higher anaerobic capacity after the 4 weeks of training. Although (–)-epicatechin did not augment the training response of the Wingate test measurements, it did not appear to inhibit any adaptations in anaerobic power as observed with relative peak VO_2_.

The current study includes limitations. A major limitation of this study is that plasma (–)-epicatechin was not measured. Although the dose administered has previously been reported to increase plasma (–)-epicatechin (34), the current study does not use the same brand of (–)-epicatechin and thus may not result in similar bioavailability. It is interesting to note that only aerobic adaptations were inhibited. Because upstream signaling mechanisms and gene expression were not investigated, it is not known what mechanism was altered to produce this response. Future research should also explore gene expression and signaling mechanisms associated with aerobic adaptations to determine if they are altered by (–)-epicatechin supplementation in conjunction with exercise training. Four weeks of training does not translate to the effects that may be observed with longer training periods. It is possible that the (–)-epicatechin group would have experienced similar increases in relative peak VO_2_ if trained over a longer period. Further, the study protocol lacked an exercise performance component such as a time-trial or time-to-exhaustion test. This is important to consider because (–)-epicatechin appeared to inhibit oxidative metabolism, despite the peak power outputs being similar. Thus, a test directly measuring performance in an endurance event could have yielded additional information on the possible physiological and/or performance benefits of (–)-epicatechin supplementation.

There is limited research involving isolated (–)-epicatechin supplementation with exercise in healthy adult populations. Research is needed to examine the effects of (–)-epicatechin on exercise performance using different dosing strategies of (–)-epicatechin. Since (–)-epicatechin is the primary flavanol found in cocoa and is usually consumed in much smaller doses, it would be beneficial to study the effects of the entire cocoa extract instead of one isolated flavanol as was used in the current study. Decroix et al. (35) found that acute supplementation of cocoa flavanols had no effect on cyclists' time trials, but did positively affect oxidative capacity in the same cyclists. It is conceivable the combination of flavonols and phytochemicals found in pure cocoa may have a different effect on skeletal muscle adaptation to exercise. Another aspect of (–)-epicatechin supplementation worthy of additional study is the timing and duration of supplementation. Instead of chronic supplementation, it may prove beneficial to examine the use of (–)-epicatechin when supplemented immediately prior to exercise. Studies have shown (–)-epicatechin increases blood flow through vasodilation by way of nitric oxide production (3). Lastly, the potential inhibition of aerobic adaptations in skeletal muscle as a result of the antioxidant properties of (–)-epicatechin need to be directly examined.

## Conclusion

Dark chocolate extracts and similar flavonol-containing extracts have been studied because of their abilities to increase exercise performance. The specific flavanol, (–)-epicatechin, has been identified to increase nitric oxide production, increase mitochondrial biogenesis, increase angiogenesis, decrease myostatin, increase follistatin, and increase exercise performance in rodents. Similar studies measuring aerobic and anaerobic capacity in humans while supplementing (–)-epicatechin are limited. The results of this study indicate no benefit of (–)-epicatechin supplementation at 200 mg per day in conjunction with cycling. Additionally, (–)-epicatechin appeared to inhibit adaptations in relative peak aerobic power and skeletal muscle SDH protein content, compared with the placebo. In conclusion, chronic (–)-epicatechin supplementation combined with exercise training in healthy adults may be disadvantageous. More research is needed to determine whether (–)-epicatechin supplementation would elicit a benefit with different dosing strategies or different exercise modalities.

## Ethics Statement

All subjects gave verbal and written informed consent in accordance with the Declaration of Helsinki. The protocol was approved by the Institutional Review Board of the University of South Alabama.

## Author Contributions

NS and ZB conceived the study idea. NS, ZB, SM-B, SH, and AW designed the research protocol. NS, ZB, SP, and SM-B conducted the data collection. NS, ZB, and SP conducted the sample analyses. NS, ZB, SP, SM-B, SH, and AW analyzed the data. NS and ZB wrote the manuscript. NS and ZB were the principal investigators and had primary responsibility for the final content. All authors read, critically revised, and approved the final manuscript.

### Conflict of Interest Statement

The authors declare that the research was conducted in the absence of any commercial or financial relationships that could be construed as a potential conflict of interest.
